# Raynaud’s phenomenon in a drummer player: Microvascular disorder and nailfold video capillaroscopic findings

**DOI:** 10.17179/excli2021-4208

**Published:** 2021-10-28

**Authors:** Maria Maddalena Sirufo, Alessandra Catalogna, Francesca De Pietro, Lia Ginaldi, Massimo De Martinis

**Affiliations:** 1 Department of Life, Health and Environmental Sciences, University of L’Aquila, 67100 L’Aquila, Italy; 2 Allergy and Clinical Immunology Unit, Center for the Diagnosis and Treatment of Osteoporosis, AUSL 04, 64100 Teramo, Italy

**Keywords:** drummer, Raynaud's phenomenon, microcirculation, arts medicine, nailfold video capillaroscopy, musician, occupational disease, hand-arm vibration syndrome

## Abstract

Drummers are usually exposed to intensive physical stress and occupational diseases that have been only partially investigated. The majority of studies focus on musculoskeletal problems while microvascular abnormalities have been less considered. We report on a case of a 19-year-old drummer affected by Raynaud's phenomenon. The patient underwent nailfold video capillaroscopy that showed a non-specific pattern, with granular flow, dyshomogeneous capillary morphology, increased efferent/afferent loop ratio and many enlarged capillaries. The continuous exposition to vibration in drummers could determinate microvascular abnormalities with related cold induced disorders and Raynaud's phenomenon. Nailfold video capillaroscopy is a tool that allows to detect the alterations of the microcirculation and to carry out the follow-up of the patients with low cost, non-invasiveness, repeatability, high sensibility and specificity.

## Introduction

Musicians are exposed to a series of occupational diseases that have been only partially investigated. The most common disorders include musculoskeletal complaints, dystonia, hearing loss and skin problems (Kok et al., 2016[8]; Enke and Poskey, 2018[6]; Sliwinska-Kowalska and Davis, 2012[18]; Crépy, 2015[4]). In clinical practice, several studies have been conducted on instruments that make up orchestras (Mizrahi, 2020[9]) such as piano or wind instruments (Sousa et al., 2017[20]; Clemente et al., 2021[3]) while much less information is available on “non-classical” musicians who play popular, rock, jazz or traditional music. In some countries, “non-classical” musicians make up the majority, in Australia for instance, 64 % of instrumentalists and singers perform contemporary music, while orchestral musicians constitute only 10 % of professional musicians (Stanhope and Weinstein, 2020[21]).

Drummers and percussionists can be placed in the category of "non-classical" musicians, and the few studies in the literature focus on musculoskeletal problems due to repetitive movements, non-physiological posture, and low and frequent joint loading; in particular, only cases of dystonia are reported in drummers (Bledsoe et al., 2021[2]). Microvascular abnormalities and Raynaud’s phenomenon (RP) have been less investigated and nailfold video capillaroscopy (NVC) is a useful instrument to detect vascular alterations in an easy and non-invasive way (Sirufo et al., 2019[17], 2020[11][14], 2021[12][16][13]; De Martinis et al., 2018[5]). 

## Case Report

**Figure 1:**
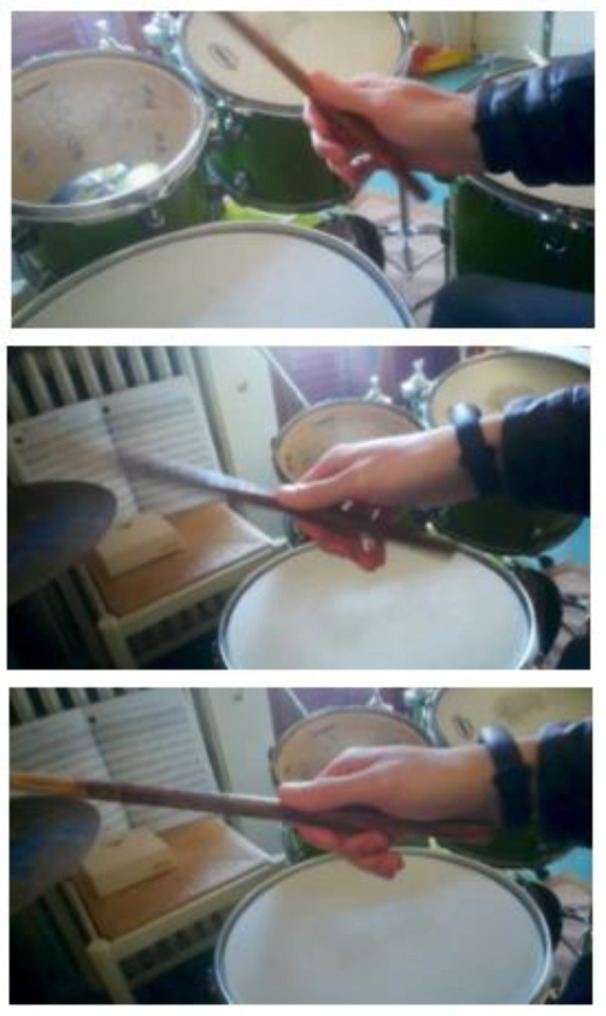
Movement description: the index finger and the thumb grasp the drumstick with a pliers grip constituting the fulcrum of the stick itself. Third and fourth fingers give the cadence of the rhythm and provide the greatest impact to the drumstick.

We report on a case of a 19-year-old musician examined for the appearance of RP in both hands and feet. This disorder started at the age of 16, increased in the winter period and when he is outdoors, it does not regress spontaneously but only if he goes to warm places. He has been playing drums since the age of 10 with the frequency of 2 times a week for 4 hours and he plays with both hands indiscriminately and also using his feet (Figure 1(Fig. 1)).

The patient went through anamnestic evaluation, physical examination and blood tests, with no relevant findings as shown in Table 1(Tab. 1). 

**Figure 2:**
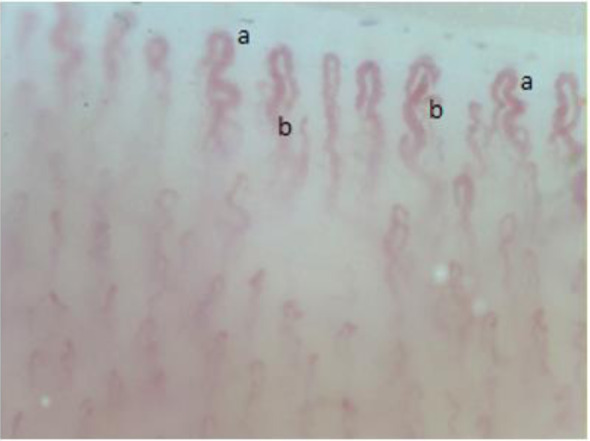
Meandering (a), dilated ratio efferent limb: afferent>2:1 (b) and ectatic “tribe clef” loops capillaries.

**Figure 3:**
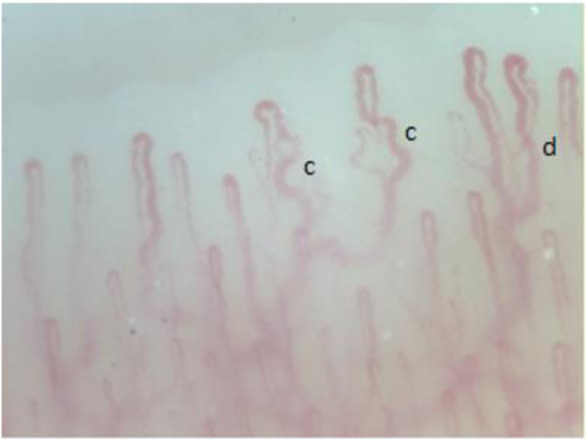
Tortuosity of the capillary (c) ectasia of the efferent tract of the loops (d).

**Figure 4:**
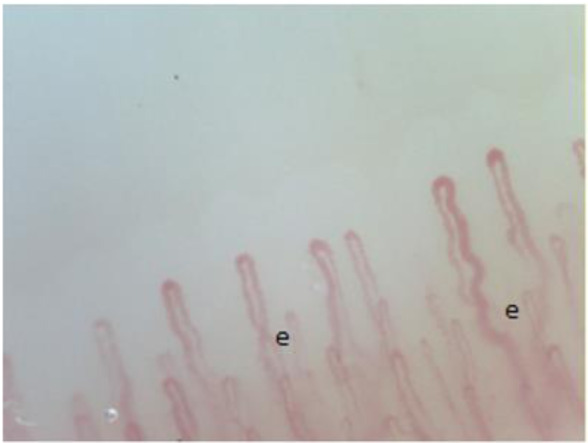
Single crossovers (e).

**Figure 5:**
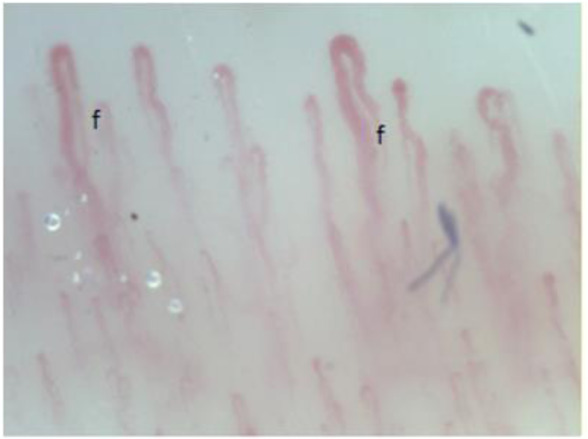
Irregular enlarged capillaries (f).

The NVC was performed using a probe equipped with 200x magnification (VideoCap software 3.0; DS Medica, Milan, Italy), in a room at a temperature of 20-25 degrees, preceded by an acclimatization of the patient. The video capillaroscope, that combines a microscope with a digital videocamera, is the gold standard device to perform NVC and it is currently considered the most suitable device for clinical and research purposes. Density and dimension (µm) of capillaries, presence of abnormal morphology and hemorrhages are the primary characteristics considered to analyze the pattern in NVC. Normal condition is featured by capillaries with convex head and hairpin shape, density ≥ 7 capillaries/mm, dimension of capillaries ≤ 20 µm, absence of abnormal morphologies and hemorrhages. A non-specific pattern is defined if any of the capillaroscopic characteristics (reduced density, dimension between 20 and 50 mm, presence of abnormal morphology and/or hemorrhages) is detected alone or in combination (Smith et al., 2020[19]). The patient underwent NVC which showed granular flow, dyshomogeneous capillary morphology, increased efferent/afferent loop ratio, many enlarged capillaries sized between 20 and 50 µm, loss of alignment and not uniform distribution of capillaries, characteristics compatible with a non-specific pattern (Figures 2-5(Fig. 2)(Fig. 3)(Fig. 4)(Fig. 5)).

This work was conducted after receiving the patient's informed consent to participate in the study and to publish this report, in compliance with the ethical standards in the field and the norms established by the Internal Review Board of University of L'Aquila (Ethics Committee of the University D.R. n. 206/2013 and D.R. n. 46/2017).

See also the Supplementary information.

## Discussion

A small number of cases of musicians with microvascular problems and RP are reported in literature, in particular two cases of guitarists (Sirufo et al., 2019[17]; Atashpaz and Ghabili, 2008[1]) and one bass player (Jepsen and Simonsen, 2016[7]). Regarding of drummers, they are continuously exposed to vibrations and the hand-arm system can suffer injuries due to the induced oscillations, in particular vascular problems such as RP. The hand-arm vibration syndrome (HAVS) is a professional syndrome characterized by an increased tendency to vasospasm in digital capillaries consisting in the disruption of the digital blood circulation and abnormal reaction to cold, known as vibration-induced white fingers, as happens in secondary RP, (Nilsson et al., 2017[10]). The HAVS is also featured by a sensory neural disorder that gradually leads to paresthesia and pain due to autonomic dysfunction and sympathetic hyperactivity (Stoyneva et al., 2003[22]).

NVC is the technique of choice for the "*in vivo*" study of the microcirculation with low cost, non-invasiveness, repeatability, high sensibility and specificity (Table 2(Tab. 2)). Little is known about how vibrations could affect the microcirculation and even less is known about some categories like musicians. Moreover, other factors such as the vibration intensity, duration of exposure, the material of the drumsticks and the way the musician holds the drumsticks itself, should be taken into account (Table 3(Tab. 3), Reference in Table 3: Welcome et al., 2015[23]).

## Conclusions

The continuous exposition to vibration to which drummers are subjected can determine microvascular abnormalities with related cold induced disorders and RP. NVC can be the instrument to diagnose these pathologies and carry out the follow-up of the patient, as well as excluding any pathologies such as connective tissue disease (Sirufo et al., 2021[12]). The diagnosis of any vascular anomalies allows the patient to have greater awareness of his or her condition and the need for a follow-up, with eventually some precautions in daily life and in the way of using the musical instrument.

Musicians are usually exposed to intense physical and physiological stresses and high levels of training from childhood, for these reasons it is important to take care of risks related to their music arts and their symptoms. NVC could have a primary role in musician health surveillance program (Sirufo et al., 2020[15]) detecting the first signs of HAVS, considering that these subjects rely on their physical and mental health to secure their professional and artistic role and even small complaints can compromise the movement sequences and musical technique creating discomfort in the artist. 

## Declaration

### Author contributions

All authors contributed equally to the work. All authors have read and agreed to the published version of the manuscript. 

### Funding

This research received no external funding. 

### Conflict of interest

The authors declare no conflict of interest. The authors declare that they have no known competing financial interest or personal relationship that could have appeared to influence the work reported in this paper.

## Supplementary Material

Supplementary information

## Figures and Tables

**Table 1:**
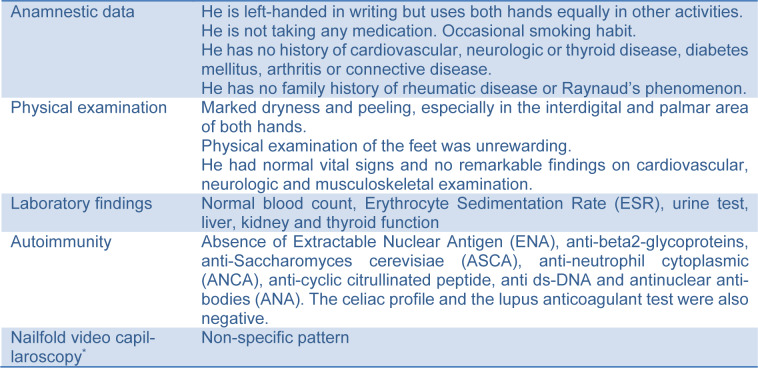
Anamnestic, clinical and laboratory features of the patient. The NVC* was conducted using a ×200 optical probe with images being captured, coded and stored using a Videocap software (DS-Medica, Milano, Italy).

**Table 2:**
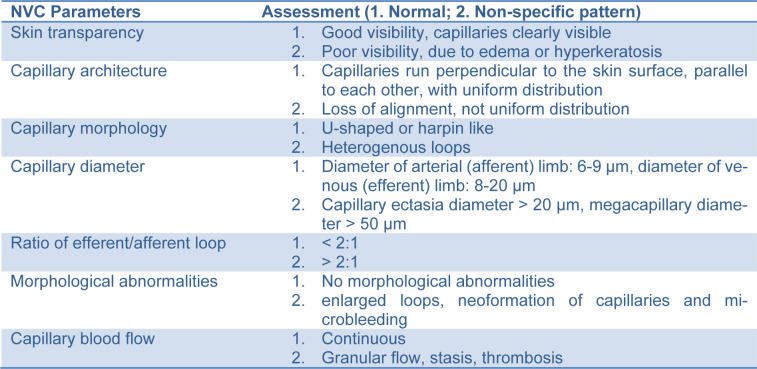
NVC parameters and assessment

**Table 3:**
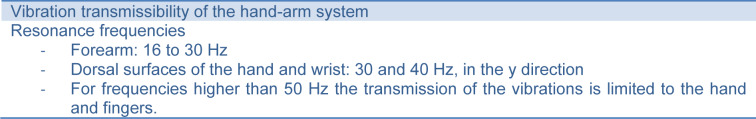
The vibration transmissibility of the hand-arm system in three orthogonal directions (Welcome et al., 2015)
