# Supplementary material for: Raynaud’s phenomenon in a drummer player: Microvascular disorder and nailfold video capillaroscopic findings
Source: EXCLI J. 2021 Oct 28;20:1526–31. doi: 10.17179/excli2021-4208 (PMC8678061; doi:10.17179/excli2021-4208)
Supplement: Supplementary information [file EXCLI-20-1526-s-001.pdf]

EXCLI Journal 2021;20:1526-1531 – ISSN 1611-2156
Received: August 17, 2021, accepted: October 21, 2021, published: October 28, 2021

Supplementary information to:

Case report:

RAYNAUD'S PHENOMENON IN A DRUMMER PLAYER: MICROVASCULAR DISORDER AND NAILFOLD VIDEO CAPILLAROSCOPIC FINDINGS

Maria Maddalena Sirufo[1,2], Alessandra Catalogna[1,2], Francesca De Pietro[1,2], Lia Ginaldi[1,2], Massimo De Martinis[1,2*]

[1] Department of Life, Health and Environmental Sciences, University of L'Aquila, 67100 L'Aquila, Italy
[2] Allergy and Clinical Immunology Unit, Center for the Diagnosis and Treatment of Osteoporosis, AUSL 04, 64100 Teramo, Italy

* **Corresponding author:** Massimo De Martinis, Department of Life, Health and Environmental Sciences, University of L'Aquila, 67100 L'Aquila, Italy.
Tel.: +39-0861-429548; Fax: +39-0861-211395; E-mail: demartinis@univaq.it

http://dx.doi.org/10.17179/excli2021-4208

This is an Open Access article distributed under the terms of the Creative Commons Attribution License (http://creativecommons.org/licenses/by/4.0/).

NAILFOLD VIDEO CAPILLAROSCOPY: STATE OF THE ART

Nailfold video capillaroscopy (NVC) is a simple, repeatable, painless and non-invasive examination widely used for the study of capillaries at the nailfold of hands because in this location the capillaries are oriented parallel to the skin surface and are generally well observable. It is performed with hand in prone position and a drop of cedar oil or paraffin is applied to the skin of the periungual area to improve the visibility of the capillaries. It is also advisable to avoid nail polish and manicures in the 20 days prior to the exam. From the 80s to today, various technologies have been developed for the visualization of capillaries, the main ones are shown in Supplementary Table 1. Currently, the most popular tool is the video capillaroscope which uses an optical probe placed in contact with the skin connected to a computer and the images of capillaries are displayed on a screen (Supplementary Figure 1) (Neubauer-Geryk et al., 2019). The magnification generally used is 200 times (200x) and it is possible to save the images. The NVC is indicated in patients with Raynaud's phenomenon for the differential diagnosis between primary (isolated) forms of Raynaud's phenomenon and those secondary to rheumatic diseases or to exposition to vibration or ionizing radiations. The NVC is widely used for the diagnostic setting of microangiopathies in the course of rheumatic and autoimmune diseases including psoriasis and psoriatic arthritis, systemic lupus erythematosus, antiphospholipid syndrome and dermatomyositis. Abnormal capillaroscopic findings are not uncommon even in individuals with conditions other than connective tissue diseases so that NVC plays a role in several other fields than rheumatology (Ciaffi et al., 2020; Sirufo et al., 2021).

EXCLI Journal 2021;20:1526-1531 – ISSN 1611-2156
Received: August 17, 2021, accepted: October 21, 2021, published: October 28, 2021

Supplementary Table 1: Different capillaroscopic devices (Riccieri et al., 2020).

	Wide-field microscopy	Ophthalmo-scopy	Dermato-scopy	Smartphone devices	Digital USB microscopy	Digital video capillaro-scopy
Image quality	Variable, depending on the im-age captur-ing system	Variable, depending on the mag-nification lens	Variable, de-pending on the magnifi-cation lens	Variable, depending on the mag-nification lens	Variable, depending on the qual-ity of the probe	Good
Costs	Medium to high	Low	Low to me-dium	Low	Low to me-dium	High
Portabil-ity	No	Yes	Yes	Yes	Yes (the probe needs to be connected to a laptop)	Yes (the probe needs to be con-nected to a laptop)
Image storage and soft-ware analysis	Possible	Not possi-ble	Possible storage but not specific software	Possible storage but not specific software	Possible	Yes
Magnifi-cation	10 to 200x	Up to 15x	Up to 10x	Up to 20x	Up to 300x	50 to 500x (200x is the most fre-quently used)
Reliabil-ity	Good	Good	Good	Good	Not tested	Good

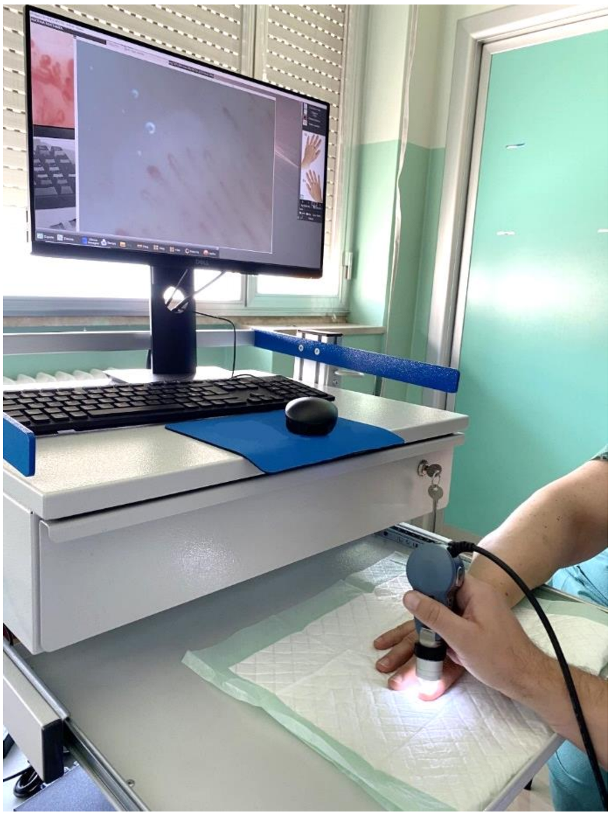

Supplementary Figure 1: Digital video capil-laroscopy: hand in prone position, a drop of cedar oil is applied to the skin of the periungual area, an optical probe is placed in contact with the skin connected to a computer, the images of the capillaries are displayed on the screen.

Received: August 17, 2021, accepted: October 21, 2021, published: October 28, 2021

This diagnostic tool allows to better understand the physiopathological aspects of microangiopathies and can be used for the staging of diseases, in particular for systemic sclerosis and for the early identification of subset of patients at higher risk of systemic complications of some rheumatic disorders (Supplementary Table 2) (Deegan and Wang, 2019). The parameters that can be evaluated are the transparency of the skin, the visibility of the venous plexus, the structure and architectural arrangement of the capillaries, the number, diameter and shape of the capillaries and the characteristics of the blood flow (Supplementary Table 3) (Karbalaie et al., 2019). The recording of clinical images through dedicated archiving software and the creation of patient personal files offers the possibility of conducting diagnostic follow-ups, comparisons between capillaroscopic pictures and teleconsultations between specialized centers, necessary to produce appropriate diagnoses. A further application of this method consists in the possibility of monitoring the effect of specific therapies by verifying the response to drugs directly at the microvascular level.

Supplementary Table 2: Principle NVC indications (Gallucci et al., 2008).

**NVC indications**	
**Rheumatic diseases**	Systemic Sclerosis
	Dermatomyositis
	Mixed Connective Tissue Disease
	Raynaud’s Phenomenon
	Undifferentiated Connective Tissue Disease
	Overlap Syndrome
	Sjögren’s Syndrome
	Systemic Lupus Erythematosus
	Antiphospholipid Antibody Syndrome
	Psoriatic Arthritis
**Non-rheumatic diseases**	Arterial Hypertension
	Diabetic Microangiopathy
	Acromegaly
	Hyper/Hypothyroidism
	Cardiac Syndrome X
	Primary Biliary-Cirrhosis
	Chron’s Disease
	Familiar Mediterranean Fever

REFERENCES

Ciaffi J, Ajasllari N, Mancarella L, Brusi V, Meliconi R, Ursini F. Nailfold capillaroscopy in common non-rheumatic conditions: A systematic review and applications for clinical practice. Microvasc Res. 2020;131:104036. doi: 10.1016/j.mvr.2020.104036.

Deegan AJ, Wang RK. Microvascular imaging of the skin. Phys Med Biol. 2019;64(7):07TR01. doi: 10.1088/1361-6560/ab03f1.

Gallucci F, Russo R, Buono R, Acampora R, Madrid E, Uomo G. Indications and results of videocapillaroscopy in clinical practice. Adv Med Sci. 2008;53:149-57. doi: 10.2478/v10039-008-0038-4.

Karbalaie A, Emrani Z, Fatemi A, Etehadtavakol M, Erlandsson BE. Practical issues in assessing nailfold capillaroscopic images: a summary. Clin Rheumatol. 2019;38:2343-54. doi: 10.1007/s10067-019-04644-9.

Neubauer-Geryk J, Hoffmann M, Wielicka M, Piec K, Kozera G, Brzeziński M, et al. Current methods for the assessment of skin microcirculation: Pt 1. Postepy Dermatol Alergol. 2019;36:247-54. doi: 10.5114/ada.2019.83656.

Riccieri V, Snow M, Stevens W, Sulli A, van Laar JM, Vonk MC, et al. Standardisation of nailfold capillaroscopy for the assessment of patients with Raynaud's phenomenon and systemic sclerosis. Autoimmun Rev. 2020;19(3):102458. doi: 10.1016/j.autrev.2020.102458.

Sirufo MM, Bassino EM, De Pietro F, Ginaldi L, De Martinis M. Nailfold capillaroscopy: Clinical practice in non-rheumatic conditions. Microvasc Res. 2021;134:104122. doi: 10.1016/j.mvr.2020.104122.

EXCLI Journal 2021;20:1526-1531 – ISSN 1611-2156
Received: August 17, 2021, accepted: October 21, 2021, published: October 28, 2021

Supplementary Table 3: Capillaroscopic characteristics (Riccieri et al., 2020).

Capillaro-scopic characteris-tics	Normal Pattern	Non-specific Pattern (At least one of the following characteristics alone or in com-bination)	Early Scleroderma Pattern	Active Scleroderma Pattern	Late Scleroderma Pattern
Density	≥ 7/mm	≤ 7/mm	≥ 7/mm	4-6/mm	≤ 3/mm
Apical diameter	≤ 20 µm	20-50 µm	> 50 µm	> 50 µm	-
Abnormal morphology	No	Yes	No	Yes (+)	Yes (++)
Hemor-rhages	No	Yes	Yes or No	Yes or No	No
	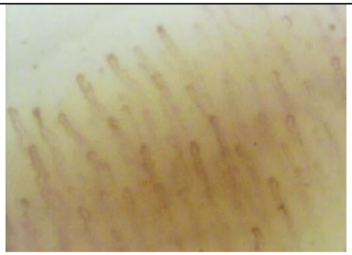	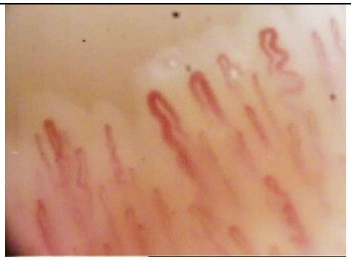	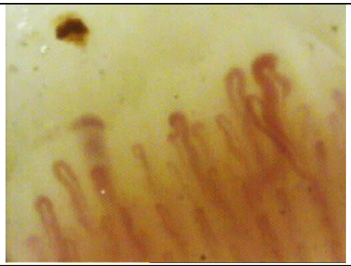	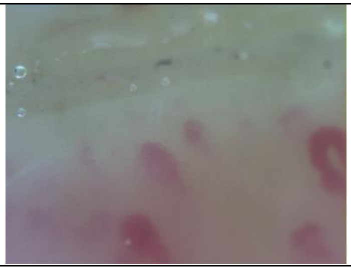	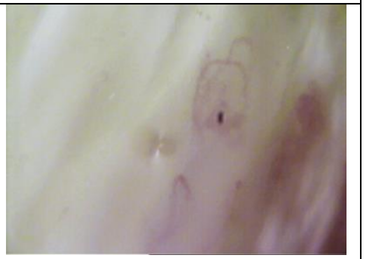
